# Prognostic significance of prognostic nutritional index and systemic immune‐inflammation index in patients after curative breast cancer resection: a retrospective cohort study

**DOI:** 10.1186/s12885-022-10218-x

**Published:** 2022-11-03

**Authors:** Tai Xu, Si-Ming Zhang, He-Ming Wu, Xiao-Min Wen, Dong-Qin Qiu, Yu-Yang Yang, Li-Zhen Wang, Wen-Biao Zhu, Li-Shan He, Jian-Juan Li

**Affiliations:** 1 Department of Breast Surgery, Meizhou People’s Hospital, No.63 Huangtang Road, Meijiang District, Meizhou, Guangdong 541000 People’s Republic of China; 2 Guangdong Provincial Key Laboratory of Precision Medicine and Clinical Translational Research of Hakka Population, Meizhou People’s Hospital, Meizhou, Guangdong People’s Republic of China; 3 Center for Precision Medicine, Meizhou People’s Hospital, Meizhou, Guangdong People’s Republic of China; 4 Clinical Laboratory Center, Meizhou People’s Hospital, Meizhou, Guangdong People’s Republic of China; 5 Department of Pathology, Meizhou People’s Hospital, Meizhou, Guangdong People’s Republic of China; 6 Clinical Pharmaceutics Room, Meizhou People’s Hospital, Meizhou, Guangdong People’s Republic of China

**Keywords:** Breast cancer, Prognostic nutritional index, Systemic immune-inflammation index, Neutrophil–lymphocyte ratio, Platelet-lymphocyte ratio

## Abstract

**Background:**

Nutritional status and inflammation are closely associated with poor outcome in malignant tumors. However, the prognostic impact of postoperative in these variables on breast cancer (BC) remains inconclusive. We aimed to determine whether prognostic nutritional index (PNI), systemic immune‐inflammation index (SII), neutrophil–lymphocyte ratio (NLR) and platelet–lymphocyte ratio (PLR) affect two long-term outcomes among patients after curative resection of BC.

**Methods:**

We retrospectively reviewed 508 patients with BC treated with curative surgery between February 5, 2013 and May 26, 2020. All patients were divided into 3 groups based on tertiles (T1-T3) of PNI, SII, NLR, and PLR. The effects of four indexes on disease-free survival (DFS) and overall survival (OS) have been evaluated using Cox proportional hazards models and Kaplan–Meier method.

**Results:**

Compared with PNI-lowest cases, patients with highest PNI showed significantly longer DFS (multivariate adjusted hazard ratio [HR] = 0.37, 95% confident interval [CI] 0.19–0.70, *P* for trend = 0.002), whereas higher PLR seemed to be marginally associated with poorer DFS (*P* for trend = 0.086 and 0.074, respectively). Subgroup analyses indicate the potential modification effects of family history of BC and radiotherapy on the prognosis value of PNI to DFS in BC patients (*P* for interaction = 0.004 and 0.025, respectively). In addition, the levels of three inflammatory indices, namely SII, NLR, and PLR might be positively related with increased age at diagnosis (all *P* for trend < 0.001).

**Conclusions:**

A high PNI was associated with better DFS, supporting its roles as prognostic parameters for patients with BC. The nutritional status and systemic immune may exert great effects on patient prognosis. Further studies are warrant to explore the prognosis value of PLR.

**Supplementary Information:**

The online version contains supplementary material available at 10.1186/s12885-022-10218-x.

## Background

Breast cancer (BC), a hormonally-dependent disease that is the primary cause of death among women worldwide [1], is a major public health problem, with 1.9 million estimated new cases worldwide with nearly 601,000 deaths in 2017 [2]. In China, more than 1.6 million people are diagnosed and 1.2 million people die of BC each year [3]. Surgical management of BC remains to be the main curative strategy against the disease in the past few years [4], and locoregional surgery for primary BC could afford an opportunity for de-escalation, with complete resection yielding the best clinical outcome [5]. Given that the recurrence rates of BC remain relatively high even after plate resection, and long-term clinical benefits like disease-free survival (DFS), and overall survival (OS) in BC patients who undergo different resection remain unclear [6], it is of great importance to investigate clinical progression among patients who underwent the surgical treatments.

The nutritional status is greatly influencing the operative complications and clinical outcomes among patients with cancer. The prognostic nutritional index (PNI), which is s simplified as a calculation of the serum albumin concertation and peripheral blood lymphocytes count, was initially designed to assess the pro-operative nutritional conditions and surgical complications among gastrointestinal cancer patients [7]. Recently, the prognostic value of PNI has been found in patients with digestive system carcinomas such as colorectal cancer [8], hepatocellular cancer [9], pancreatic cancer [10], and gastric cancer [11]. However, the impact of PNI on the long-term outcomes in patients with BC remains controversial [12, 13].

In addition, inflammation has been demonstrated to be associated with the increased risk of cancers due to its characteristics in tumorigenesis, progression, and metastasis [14]. The incorporation of biochemical markers of inflammatory response in prognostic scores for several types of cancers has been reported [15]. Biochemical parameters such as white cells, neutrophil counts, and hypoalbuminemia are evaluated in cancer patients [16]. Platelets also play a special role in the inflammatory response and thrombocytosis which are not uncommon in patients with solid tumors [17]. The prognostic values of inflammatory biomarkers like systemic immune‐inflammation index [SII], which is calculated by the counts of platelet, neutrophil, and lymphocyte in peripheral blood, were recommended in the development of cancer [18]. A recent meta-analysis involving 2,642 patients consistently indicated that elevated SII predicts poor survival outcomes [19].

Similarly, the neutrophil–lymphocyte ratio (NLR) and platelet–lymphocyte ratio (PLR), two representative blood markers of the systemic inflammatory response, are useful to predict the prognoses in various malignancies [20, 21]. A meta-analysis including 7,951 patients from 12 studies showed that BC patients with a higher NLR had a shorter DFS (hazard ratios [HRs] = 1.46, 95% confident intervals [95%CIs]: 1.12–1.90, *P* = 0.044) and OS (HR = 2.03, 95%CI: 1.41–2.93, *P* < 0.001) [22]. High PLR was also found to be significantly associated with poor disease-specific survival [DSS] and DFS in breast cancer patients, with HRs and their 95%CIs, were 3.23 (1.77–5.89) for DSS and 1.82 (1.82–6.32) for DFS [23]. However, the predictive significance of systemic inflammation-based prognostic indices that combine SII, NLR, and PLR have not attached much research attention in patients with BC.

Accordingly, the purpose of this study was to investigate the associations between PNI, SII, NLR, and PLR and two long-termed clinic outcomes (OS and DFS) in patients who underwent surgical resections of BC.

## Methods

### Setting and participants

Eligible patients were enrolled at Meizhou People’s hospital between February 5, 2013, and May 26, 2020. Inclusion criteria for this study were: i) Age was ≥ 18 years; ii) BC patients who were undergoing curative resection; iii) patients agreed to participate and signed informed consent. We excluded patients if i) they aged under 18 years; ii) there were missing medical data and laboratory results before the surgery; iii) they had not undergone surgical resection, or iv) there were missing or implausible data on clinical tests during follow‐up. The institutional review board and medical ethics committee of Meizhou People’s hospital tested and approved the research protocol of this study, which was conducted in compliance with the Declaration of Helsinki. Written consent was obtained from all subjects and/or their legal guardian(s).

### Data collection

Demographic, clinical, and histopathological data such as a family history of BC, body mass index (BMI), age at diagnosis, date of diagnosis, the preoperative and pro-operative pathological diagnosis of tumor, clinical stage of the tumor, tumor size, estrogen receptor (ER), progesterone receptor (PR), human epidermal growth factor receptor type 2 (HER-2) status, Ki67 index, surgical form, dates, and types of treatments (chemotherapy, radiotherapy, endocrine therapy and targeted therapy), survival status, date and location of lymph node metastasis (LNM) were obtained from the patient clinical reports [24]. Laboratory data collected and tested within one week before surgery such as basal‐pretherapeutic blood cytology and blood chemistry were obtained from the hospital information operating system.

### Definitions and primary outcomes

All patients had clinical follow-ups by the same team of surgeons. Patients were regularly followed up as outpatients every 1 to 3 months after discharge until the last day of follow-up or death. The PNI, SII, NLR and PLR were calculated using the following formulas: PNI = serum albumin level (g/dL) × 10 + lymphocyte count in peripheral blood (/nL) × 0.005; SII = platelet (P) × neutrophil (N) / lymphocyte counts (L); NLR = absolute neutrophil count / absolute lymphocyte count; and PLR = absolute platelet count / absolute lymphocyte count, respectively [7, 18, 23]. The definition of OS refers to the period between the date of surgery and the date of death [25], and DFS was defined as the time elapsed between the date of surgery and the date of the last follow-up or tumor recurrence, whichever came first [26].

### Statistical analyses

Data were expressed as frequency and percentage for categorical variables, and abnormally distributed quantitative variables were analyzed as median and inter-quantile range (IQR). PNI, SII, NLR, and PLR were categorized by tertiles; trends across tertiles were analyzed by general linear regression for continuous variables, and by Cochran-Armitage (CA) test for categorical variables. Cox proportional hazard analysis in univariate and multivariate models was performed to compare HRs and their 95%CIs according to the tertiles of four indexes. A similar method described above was used as the primary analysis to assess the effects of four indexes on patient survival across the following subgroups: family history of BC (No vs. Yes), BMI (18.5–22.9 kg/m2, 23–27.4 kg/m2, 27.5 kg/m2), surgical forms (breast-conserving surgery, modified radical mastectomy, total mastectomy, and unclear), pathological diagnosis (invasive carcinoma *vs.* carcinoma in situ), clinical-stage (0, I, II, III, IV), tumor size (≥ 4 cm *vs.* < 4 cm), status of ER (negative *vs.* positive), status of PR (negative *vs.* positive), status of HER-2 (negative *vs.* positive), Ki67-index (≥ 27.5% *vs.* < 27.5%), chemotherapy (No *vs.* Yes), radiotherapy (No *vs.* Yes), endocrine therapy (No *vs.* Yes), target therapy (No *vs.* Yes), and LNM (No *vs.* Yes) were analyzed by subgroup analyses in the multivariable-adjusted model. The survival time graphs were evaluated using the Kaplan–Meier methods, and compared using log-rank tests. All tests were two-sided, and a 2-sided α < 0.05 was considered statistically significant. All statistical analyses were performed using the R software version 3.5.1.

## Results

### Patient and tumor characteristics

A total of 508 patients were enrolled and followed up until June 30, 2020, and their clinicopathological features are given in Table 1. The median age at diagnosis of BC was 49.0 years (IQR: 43.0–57.0). The medians and IQRs of PNI, SII, NLR, and PLR were 55.2 (6.7), 514.3 (411.9), 2.0 (1.3), and 131.4 (56.7), respectively. As for the period of survival, the median DFS and OS were 1393.0 days (IQR: 485.0–1695.5) and 1480.0 days (IQR: 689.5–1728.0), respectively. Among 508 included patients, 367 (72.24%) patients did not report a positive family history of BC, and 14.76% (75/508) of them had BMI larger than 27.5 kg/m2. 452 (88.98%) patients were diagnosed with invasive carcinoma. According to the TNM classification staging system [27], 37 (7.28%) patients were classified in stage 0, 107 (21.06%) in stage I, 263 (51.77%) in stage II, 100 (19.69%) in stage III, and only 1 (0.20%) in stage IV. 391 (76.97%) patients have larger tumor size (≥ 4.0 cm). 357 (70.28%), 303 (59.65%), and 347(68.31%) patients reported positive results in ER, PR and HER-2, respectively. 53.54% (2772/508) of patients reported Ki67-index up to 27.5%. Of four surgery forms, 61 (12.01%) patients chose the breast-conserving surgery, 249 (49.02%) patients conducted the modified radical mastectomy, and 190 (37.40%) patients performed the total mastectomy, with unclear data on surgery methods of 8 (1.57%) patients. As for other treatment methods, 368 (72.44%), 153 (30.12%), 348 (68.50%), and only 46 (9.06%) patients have conducted the chemotherapy, radiotherapy, endocrine therapy, and targeted therapy, respectively. The proportions of patients with and without clinic LNM were 43.90% (223/508) and 55.71% (283/508).

**Table 1:** Baseline characteristics (*n* = 508)

Variables	Total	PNI			SII			NLR			PLR		
		**T1**	**T3**	***P*** ^a^	**T1**	**T3**	***P*** ^a^	**T1**	**T3**	***P*** ^a^	**T1**	**T3**	***P*** ^a^
**Age (IQR)**^b^	49.0 (43.0– 57.0)	48.0 (43.5– 55.0)	50.0 (43.0– 58.0)	0.970	52.0 (44.0. 59.0)	46.0 (42.0– 51.0)	< 0.001	52.0 (45.0– 59.0)	46.0 (43.0– 52.0)	< 0.001	51.0 (44.0– 60.0)	47.0 (43.0– 52.0)	** < 0.001**
**DFS, day (IQR)**^b^	1393.0 (485.0– 1695.5)	811.0 (326.0– 1548.5)	1589.0 (1324.5– 1768.0)	< 0.001	1409.0 (484.0– 1671.8)	1338.0 (419.0– 1673.5)	0.212	1414.0 (548.8– 1679.5)	1344.0 (419.0– 1677.0)	0.173	1435.5 (637.8– 1723.0)	1246.0 (427.0– 1585.0)	**0.039**
**OS, day (IQR)**^b^	1480.0 (689.5– 1728.0)	1029.0 (340.0– 1614.0)	1609.0 (1393.5– 1774.5)	< 0.001	1483.5 (730.8– 1709.3)	1397.5 (490.3– 1719.8)	0.206	1476.0 (724.3– 1705.0)	1454.0 (515.0– 1715.0)	0.461	1510.0 (731.5– 1758.8)	1383.0 (465.5– 1653.3)	**0.047**
**Family history of BC**				0.070			0.715			0.814			0.543
No	367 (72.24)	117 (23.08)	129 (25.44)		120 (23.86)	123 (24.45)		126 (24.95)	119 (23.56)		124 (24.65)	119 (23.66)	
Yes	141 (27.76)	54 (10.65)	38 (7.50)		48 (9.54)	45 (8.95)		48 (9.50)	48 (9.50)		44 (8.75)	49 (9.74)	
**Body mass index (kg/m2)**				0.675			0.326			0.341			0.114
18.5–22.9	168 (33.07)	53 (10.45)	60 (11.83)		58 (11.53)	55 (10.93)		56 (11.09)	54 (10.69)		51 (10.14)	56 (11.13)	
23–27.4	236 (46.46)	79 (15.58)	82 (16.17)		73 (14.51)	74 (14.71)		82 (16.24)	76 (15.05)		84 (16.70)	68 (13.52)	
≥ 27.5	75 (14.76)	30 (5.92)	18 (3.55)		29 (5.77)	24 (4.77)		31 (6.14)	25 (4.95)		30 (5.96)	28 (5.57)	
**Histological diagnosis**				0.657			0.603			0.368			0.623
Invasive carcinoma	452 (88.98)	151 (29.78)	150 (29.59)		146 (29.03)	149 (29.62)		150 (29.70)	149 (29.50)		150 (29.82)	153 (30.42)	
Carcinoma in situ	56 (11.02)	20 (3.94)	17 (3.35)		22 (4.37)	19 (3.78)		24 (4.75)	18 (3.56)		18 (3.58)	15 (2.98)	
**Clinical stage**				0.848			0.787			0.878			0.757
0	37 (7.28)	11 (2.17)	10 (1.97)		13 (2.47)	14 (2.75)		13 (2.47)	19 (2.61)		12 (2.34)	11(2.20)	
I	107 (20.06)	34 (6.69)	35 (6.89)		39 (7.75)	36 (7.15)		35 (6.95)	33 (6.56)		33 (6.56)	39 (7.75)	
II	263 (51.77)	92 (18.11)	89 (17.52)		87 (17.04)	89 (17.47)		92 (18.11)	81 (17.26)		93 (18.32)	79 (15.55)	
III	100 (19.69)	32 (6.30)	35 (6.89)		32 (6.21)	25 (4.92)		35 (6.85)	28 (5.99)		33 (6.42)	35 (6.85)	
IV	1 (0.20)	1 (0.20)	0 (0.00)		0 (0.00)	1 (0.10)		0 (0.00)	0 (0.00)		0 (0.00)	1 (0.11)	
**Tumour size, cm**				0.019			0.765			0.646			0.293
≥ 4	391 (76.97)	123 (24.26)	139 (27.42)		136 (27.04)	133 (26.44)		141 (27.92)	127 (25.15)		128 (25.45)	136 (27.04)	
< 4	79 (15.55)	35 (2.56)	19 (1.78)		23 (4.57)	20 (3.98)		22 (4.36)	23 (4.55)		29 (5.77)	22 (4.37)	
**Status of ER**				0.329			0.632			0.421			0.657
Negative	149 (29.33)	46 (9.07)	53 (10.45)		48 (9.54)	52 (10.34)		50 (9.90)	55 (10.89)		48 (9.54)	52 (10.34)	
Positive	357 (70.28)	124 (24.46)	113 (22.29)		119 (23.66)	115 (22.86)		123 (24.36)	112 (22.18)		119 (23.66)	116 (23.06)	
**Status of PR**				0.859			0.436			0.964			0.947
Negative	203 (39.96)	68 (13.41)	68 (13.41)		68 (13.52)	61 (12.13)		68 (13.47)	66 (13.07)		66 (13.13)	67 (13.32)	
Positive	303 (59.65)	102 (20.12)	98 (19.33)		99 (19.68)	106 (21.07)		105 (20.79)	101 (20.00)		101 (20.08)	101 (20.08)	
**Status of HER-2**				0.175			0.568			0.448			0.311
Negative	114 (22.44)	33 (6.51)	43 (8.48)		41 (8.15)	34 (6.76)		47 (9.31)	38 (7.52)		43 (8.55)	33 (6.56)	
Positive	347 (68.31)	122 (24.06)	109 (21.50)		116 (23.06)	113 (22.47)		115 (22.77)	112 (22.18)		113 (22.47)	115 (22.86)	
**Ki67-index**^c^				0.186			0.229			0.122			0.827
≥ 27.5%	272 (53.54)	84 (16.57)	94 (18.54)		98 (19.48)	87 (17.30)		101 (20.00)	83 (16.44)		90 (17.89)	92 (18.29)	
< 27.5%	236 (46.46)	87 (17.16)	73 (14.40)		70 (13.92)	81 (16.10)		73 (14.46)	77 (16.63)		78 (15.51)	76 (15.11)	
**Surgical forms**				0.979			0.209			0.763			0.202
Breast conserving surgery	61 (12.01)	20 (3.87)	21 (4.07)		17 (3.29)	26 (5.04)		24 (4.65)	23 (4.45)		16 (3.10)	26 (5.04)	
Modified radical mastectomy	249 (49.02)	80 (15.68)	83 (16.27)		82 (16.07)	72 (14.09)		91 (17.86)	76 (14.88)		89 (17.46)	82 (16.07)	
Total mastectomy	190 (37.40)	70 (13.73)	61 (11.94)		68 (13.33)	66 (12.93)		59 (11.54)	65 (12.73)		63 (12.33)	56 (10.94)	
Unclear	8 (1.57)	3 (0.57)	3 (0.57)		2 (0.43)	4 (0.86)		1 (0.29)	4 (0.72)		1 (0.29)	4 (0.86)	
**Chemotherapy**				0.473			0.464			0.994			0.144
No	140 (27.56)	49 (9.66)	42 (8.28)		51 (10.14)	45 (8.95)		48 (9.50)	46 (9.11)		53 (10.54)	41 (8.15)	
Yes	368 (72.44)	122 (24.06)	125 (24.65)		117 (23.26)	123 (24.45)		126 (24.95)	121 (23.96)		115 (22.86)	127 (25.25)	
**Radiotherapy**				0.570			0.635			0.714			0.343
No	355 (69.88)	119 (23.47)	121 (23.86)		118 (23.46)	114 (22.66)		123 (24.36)	115 (22.77)		120 (23.86)	112 (22.27)	
Yes	153 (30.12)	52 (10.26)	46 (9.07)		50 (9.94)	54 (10.74)		51 (10.10)	52 (10.30)		48 (9.54)	56 (11.13)	
**Endocrine therapy**				0.618			0.412			0.339			0.410
No	160 (31.50)	52 (10.26)	55 (10.85)		50 (9.94)	57 (11.33)		54 (10.69)	60 (11.88)		52 (10.34)	59 (11.73)	
Yes	348 (68.50)	119 (23.47)	112 (22.09)		118 (23.46)	111 (22.07)		120 (23.76)	107 (21.19)		116 (23.06)	109 (21.67)	
**Targeted therapy**				0.380			0.566			0.758			0.848
No	462 (90.94)	155 (30.57)	156 (30.77)		149 (29.62)	152 (30.22)		161 (31.88)	153 (30.30)		153 (30.42)	154 (30.62)	
Yes	46 (9.06)	16 (3.16)	11 (2.17)		19 (3.78)	16 (3.18)		13 (2.57)	14 (2.77)		15 (2.98)	14 (2.78)	
**LNM, clinical**				0.902			0.501			0.188			0.789
No	283 (55.71)	99 (18.15)	92 (18.15)		97 (19.28)	102 (20.28)		92 (18.22)	99 (19.60)		94 (18.69)	91 (18.09)	
Yes	223 (43.90)	77 (15.19)	75 (14.79)		71 (14.12)	64 (12.72)		82 (16.24)	66 (13.07)		74 (14.71)	76 (15.11)	

*Abbreviations*: *PNI* Prognostic nutritional index, *SII* Systemic immune‐inflammation index, *NLR* Neutrophil–lymphocyte ratio, *PLR* Platelet-lymphocyte ratio, *IQR* Inter-quartile range, *BC* Breast cancer, *T* Tertile, *LNM* Lymph node metastasis, *ER* Estrogen receptor, *PR* Progesterone receptor, *HER-2* Human epidermal growth factor receptor type 2, *Ki67 index* Percentage of Ki67-positive cancer nuclei, *DFS* Disease-free survival, *OS* Overall survival

^a^*P*, *P* for trend

^b^Skewered distributed data was described as median and IQR

^c^Cut-off value of 27.5% was calculated by the ROC curve

Patient characteristics stratified by tertiles of PNI, SII, NLR, and PLR are also summarized in Table 1. Patients with indexes values of ≤ 53.0, 53.0 ~ 57.5, > 57.5 for PNI, ≤ 429.43, 429.43 ~ 665.24, and > 665.24 for SII, ≤ 1.71, 1.71 ~ 2.43, and > 2.43 for NLR, ≤ 115.66, 115.66 ~ 150.65, > 150.65 for PLR were divided into tertile 1 (T1, lowest), tertile 2 (T2), and tertile 3 (T3, highest), respectively. The median age at diagnosis significantly decreased as SII, NLR, and PLR tertiles increased (all *P* for trend < 0.001), and no significant trend was observed for PNI (*P* for trend = 0.970). Both DFS and OS significantly increased as PNI tertile increased (DFS: T1: 811.0 (326.0–1548.5) *vs.* T3: 1589.0 (1324.5–1768.0);OS: T1: 1029.0 (340.0–1614.0) *vs.* T3: 1609.0 (1393.5–1774.5); both *P* for trend < 0.001), whereas both DFS and OS significantly decreased as PLR tertile increased (DFS: T1: 1435.5 (637.8–1723.0) *vs.* T3: 1246.0 (427.0–1585.0);OS: T1: 1510.0 (731.5–1758.8) *vs.* T3: 1383.0 (465.5–1653.3); both *P* for trend < 0.05), and no significant trend was observed across tertiles of SII and NLR (both *P* for trend > 0.05).The percentage of those with larger tumor size (≥ 4 cm) significantly increased across tertiles of PNI (*P* for trend = 0.019), while no significant trend was found across tertiles of SII, NLR, and PLR (*P* for trend ranged from 0.293 to 0.765).

### Relationship between inflammation‐based biomarkers and patient survival

As it shown in Table 2, univariate Cox proportional hazard analyses showed that patients in the highest category of PNI (> 57.5) high PNI was associated with better DFS (rude HR = 0.41, 95%CI: 0.22–0.75; *P* = 0.004; *P* for trend = 0.004) compared to those in lowest category (≤ 53.0). By contrast, patients in the second exposure level of PLR (115.66 ~ 150.65) experienced worse DFS (rude HR = 2.00, 95%CI: 1.05–3.81; *P* value = 0.036) compared with the reference group (≤ 115.66), despite the test for trend did not reach significance (*P* for trend = 0.086).

**Table 2:** Univariate and multivariate analyses for DFS and OS in BC patients with PNI, SII, NLR and PLR

	**DFS**					**OS**				
	**HR**	**95% CI**		***P* value**	***P* trend**	**HR**	**95% CI**		***P* value**	***P* trend**
**Univariate analyses**										
**PNI**					0.004					0.294
T1	1.00	1.00	1.00	-		1.00	1.00	1.00	-	
T2	0.55	0.31	0.98	0.044		0.38	0.10	1.54	0.177	
T3	0.41	0.22	0.75	0.004		0.52	0.16	1.70	0.278	
**SII**					0.949					0.663
T1	1.00	1.00	1.00	-		1.00	1.00	1.00	-	
T2	1.59	0.89	2.86	0.119		1.45	0.35	6.09	0.609	
T3	1.01	0.52	1.96	0.977		1.41	0.31	6.28	0.656	
**NLR**					0.316					0.931
T1	1.00	1.00	1.00	-		1.00	1.00	1.00	-	
T2	1.09	0.59	2.00	0.794		1.21	0.32	4.51	0.777	
T3	1.35	0.75	2.45	0.318		1.06	0.27	4.24	0.933	
**PLR**					0.086					0.196
T1	1.00	1.00	1.00	-		1.00	1.00	1.00	-	
T2	2.00	1.05	3.81	0.036		0.47	0.09	2.59	0.389	
T3	1.83	0.94	3.55	0.076		2.11	0.62	7.24	0.233	
**Multivariate analyses**^a^										
**PNI**					0.002					0.399
T1	1.00	1.00	1.00	-		1.00	1.00	1.00	-	
T2	0.49	0.26	0.91	0.024		0.46	0.11	2.02	0.304	
T3	0.37	0.19	0.70	0.002		0.54	0.14	2.05	0.369	
**SII**					0.629					0.686
T1	1.00	1.00	1.00	-		1.00	1.00	1.00	-	
T2	1.67	0.90	3.08	0.101		1.09	0.23	5.06	0.917	
T3	0.80	0.39	1.65	0.548		1.40	0.27	7.18	0.684	
**NLR**					0.350					0.872
T1	1.00	1.00	1.00	-		1.00	1.00	1.00	-	
T2	1.33	0.69	2.58	0.395		0.87	0.20	3.76	0.852	
T3	1.37	0.71	2.62	0.345		0.89	0.20	3.91	0.876	
**PLR**					0.074					0.208
T1	1.00	1.00	1.00	-		1.00	1.00	1.00	-	
T2	2.40	1.19	4.81	0.014		0.23	0.03	2.15	0.200	
T3	1.98	0.95	4.10	0.067		2.32	0.63	8.55	0.207	

T1-T3, PNI were divided into 3 quartiles: ≤ 53.0, 53.0 ~ 57.5, > 57.5; SII were divided into 3 quartiles: ≤ 429.43, 429.43 ~ 665.24, > 665.24; NLR were divided into 3 quartiles: ≤ 1.71, 1.71 ~ 2.43, > 2.43; PLR were divided into 3 quartiles: ≤ 115.66, 115.66 ~ 150.65, > 150.65

*Abbreviations*: *DFS* Disease-free survival, *BC* Breast cancer, *HR* Hazard ratio, *CI* Confident interval, *PNI* Prognostic nutritional index, *SII* Systemic immune‐inflammation index, *NLR* Neutrophil–lymphocyte ratio, *PLR* Platelet-lymphocyte ratio, *T* Tertile, *P P* for trend

^a^Multivariate model is adjusted for age, surgical options, pathological diagnosis; lymph node metastasis; tumour size; status of ER; status of PR; clinical classification; chemotherapy; radiotherapy; endocrine therapy; targeted therapy; family history of breast cancer

**Fig. 1:**
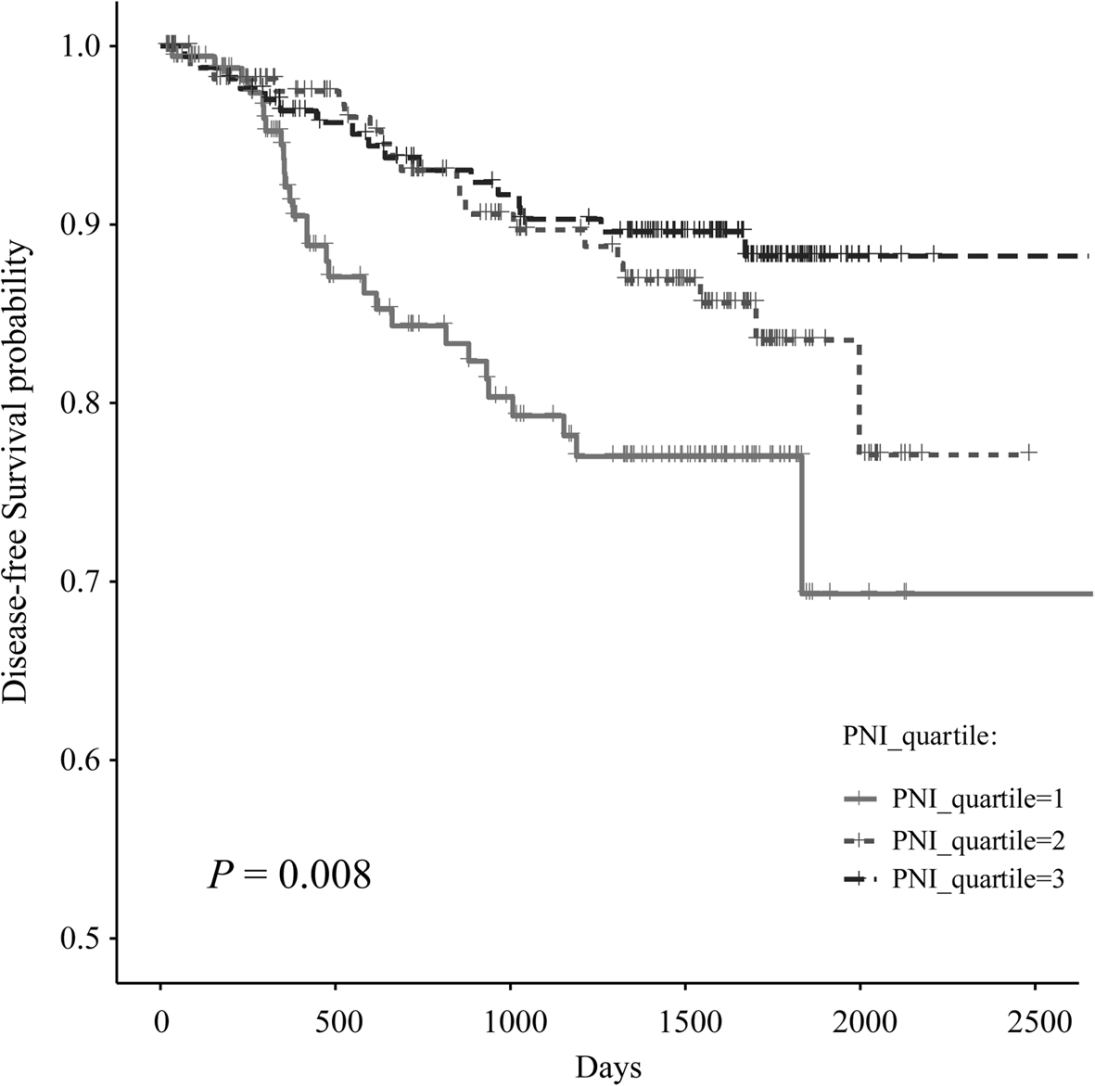
The Kaplan‐Meier disease-free survival curves of all patients in the cohort according to tertiles of PNI (log-rank analysis *P* < 0.05)

Consistently, in multivariate analysis, patients in the high PNI group enjoyed better DFS compared to those in the lowest PNI group (adjusted HR = 0.37, 95%CI: 0.19–0.70; *P* = 0.002; *P* for trend = 0.002), whereas patients who showed second PLR level before surgery might have significantly worse DFS compared with those who in lowest PLR level (adjusted HR = 2.40, 95%CI: 1.19–4.81; *P* = 0.014; *P* for trend = 0.074). On the contrary, no matter in the univariate or multivariate model, null associations between SII or NLR were observed (all *P* for trend > 0.05), as well as associations between PNI, SII, NLR and PLR and OS (*P* for trend ranged from 0.196 to 0.931 in two models). In the Kaplan–Meier analysis, the highest PNI group showed a significantly better DFS (log-rank *P* = 0.008) than the lowest PNI group (Fig. 1).

Given that a null association between four indexes and OS was observed, several pre-specified subgroup analyses were only performed to explore the prognostic impact of four indexes on DFS in BC patients after curative surgery (supplementary tables 1, 2, 3 and 4; supplementary figures 1, 2 and 3). As presented in supplementary table 1, among patients without a family history of BC, patients in the high PNI group had a better DFS compared to those in the low group (*P* for interaction = 0.004). Besides, in those who had conducted radiotherapy, DFS was still significantly longer in the highest PNI group than in the lowest PNI group (*P* for interaction = 0.025). Of note, results of several pre-specific subgroups (clinical stage, surgical forms, and targeted therapy) failed to be evaluated or were hampered by small sample sizes and low statistical power. Therefore, nonsignificant findings in these subgroups need further investigation.

## Discussion

Our findings indicate that higher PNI was significantly associated with better DFS, whereas higher PLR seemed to be associated with poorer DFS. Subgroup analyses showed that the benefits of high PNI to DFS were more evident in those without a family history of BC, as well as in those who have conducted the radiotherapy. In addition, the levels of SII, NLR, and PLR might be positively associated with increased age at diagnosis.

The PNI is a surrogate marker for the nutritional and immunological statuses in patients and has been known as an important prognostic factor in several digestive system carcinomas (e.g., colorectal cancer, hepatocellular cancer, pancreatic cancer, and gastric cancer) [8–11]. Prior studies have also assessed the prognostic value of PNI among patients with BC. For instance, in a retrospective study, Hua X, et al*.* found that the OS of patients in the higher PNI group (> 52.0) was significantly better than those in the lower PNI group (≤ 52.0) [28]. Similarly, in a latest retrospective study, Chen L, et al*.* consistently found that patients with higher pre-operative PNI value was associated prolonged DFS and OS compared to those with lower PNI (DFS: 47.64 *vs.* 36.60 months, *P* < 0.001, HR = 0.26 (95%CI = 0.16–0.44); OS: 73.61 *vs.* 64.97 months, *P* < 0.001, HR = 0.32 (95%CI = 0.21–0.49)) [29]. However, their findings might not be generalized for all types of BC or those who conducted other surgical treatments because the study by Hua X, et al*.* [28] has only been conducted among BC patients who conducted in T1-2N1 (invasive BC and no distant metastasis] female BC patients who received a radical or modified radical mastectomy, with those who received breast-conserving surgery excluded. The study by Chen L, et al*.* only enrolled female BC patients who had undergone neoadjuvant chemotherapy followed by operation, and BC patients who received anti-inflammatory medications (chemotherapy, radiotherapy, endocrine therapy, targeted therapy, immunotherapy, etc.) and those with synchronous and metachronous tumors or distant metastases have been excluded in their study [29]. In contrast to the above investigations, our study was conducted among female BC patients without specific limitations of clinical diagnosis, surgery methods, or other treatments, and this could not only ensure the generalization of our findings to a certain extent but also enable us to evaluate the modification effects of surgery methods as well as other treatments.

Due to the simplification of the calculation formula of PNI, it has become one of the most easily measured and widely used indicators in routine clinical practice. However, the mechanism by which a low PNI decreases the survival time of patients with BC has not been fully elucidated. The PNI is based on lymphocyte count and albumin level, which are significantly closely related to the prognosis of cancer patients [30], and a low PNI indicates increased inflammatory reaction and poor nutritional status [31]. Prior study reported that BC patients with higher serum albumin showed 45% reduced risk of death (HR = 0.55, 95% CI: 0.40–0.75) than those with lower albumin level, indicating that albumin could be an independent predictor of prognosis of BC patients [32]. Additionally, the lymphocyte level is an indicator of cell-mediated immunity and could exert a great effect on the adaptive immune system which regulates the growth of tumor cells [33]. The subpopulations of lymphocyte cells such as CD4 + and CD8 + T‐cells, gamma‐delta T‐cells, natural killer (NK) cells, and B‐cells have also been known to regulate the tumor progression [34]. Of note, malnutrition could further weaken the immune function, resulting in increased side effects, affecting the sensitivity of radiotherapy or chemotherapy, and lowering the intensity of chemotherapy [28]. This means that the low sensitivity of radiotherapy or chemotherapy of BC patients with low PNI value might result in impaired immune function, then lead to worse survival [28].

By contrast, the PLR, a marker of systemic inflammation, seemed to be associated with poor prognosis in BC patients. An increasing number of evidence have indicated that PLR was linked to poor prognosis in several malignancies. For example, a meta-analysis involving 11 articles reported the association between elevated PLR and decreased OS (HR = 1.33, 95% CI: 1.10–1.62) among patients with lung cancer [35]. A meta-analysis involving 20 studies with 12,754 patients showed that high PLR was significantly associated with worse OS among patients with colorectal, gastroesophageal, hepatocellular, pancreatic, and ovarian cancers (HRs ranged from 1.57 to 3.33), but not in those with BC (HR = 1.60, 95%CI: 0.59–4.34) [20]. However, another meta-analysis study reported the association between high PLR levels and poor DFS and OS among BC patients, with their HRs and 95%CIs, were 1.47 (95% CI: 1.16–1.85) for DFS, and 1.88 (95% CI: 1.27–2.80) for OS, respectively [36]. Although the mechanisms by which the PLR impacts the prognosis of patients with cancer have not been greatly understood, previous studies indicated that platelets not only could contribute to tumor growth, invasion, and angiogenesis, but also facilitate tumor metastasis by promoting tumor cells from natural killer cell-mediated lysis [37, 38]. Given that both results of PLR in univariate and multivariate models were just marginally significance, more studies are needed to further explore the prognostic value of PLR to BC.

In addition, we found that the levels of three inflammation-based prognostic scores, namely SII, PLR, and NLR, increased as the age at diagnosis increased (all *P* for trend < 0.001). The previous study consistently found that both medians of PLR and NLR were significantly higher among those aged 65 and above compared to those aged between 18 and 65 (PLR: 139 (IQR: 226–169) *vs.* 106 (IQR: 88–128); NLR: 5.00 (IQR: 4.33, 8.00) *vs.*4.83 (IQR: 4.00- 6.00); both *P* < 0.001) [39]. This might be partly attributable to age-related chronic inflammation since many inflammatory cytokines including serum levels of interleukin-6 [IL-6], tumor necrosis factor-α [TNFα], and IL-18 tend to increase among the elderly population. Evidence indicates that mechanisms that limit the basal inflammation would be dysregulated during aging, although microbial infection is required in most acute inflammation. Further studies are warranted on the mechanisms of age-related inflammation.

The subgroup analyses indicate that the prognosis value of PNI to DFS was more evident among BC patients without a family history of BC and those who had received radiotherapy (both *P* for interaction < 0.05). Given that a family history of breast cancer has been identified as one of the traditional risk factors for BC [40], further studies are needed to clarify the effect of modification of a family history of BC on the association between PNI and clinic outcomes of BC. Besides, Hua X, et al*.* also found that the association between high-PNI and longer OS was much evident in patients who had conducted radiotherapy [28]. It is well known that radiotherapy could reduce the local recurrence of BC risk thus improving survival among patients with advanced stages of tumor [41]. The immune function of patients with high PNI might be better due to their better sensitivity to radiotherapy [28].

Our study had several limitations. Firstly, this is a single-center study and our findings might not be generalized to other populations. Secondly, confirmatory conclusions cannot be drawn from the results of the current study due to its retrospective feature. Thirdly, four indexes were calculated according to the pre-operative blood sample, and this limited us to consider other factors such as changes in hemodynamic and physiology changes that can occur during surgery and in the early postoperative period [42]. Finally, due to the nature of retrospective study, the protocols for radiotherapy vary from person to person and cannot be further analyzed in the subgroup analyses.

## Conclusion

Our study shows that preoperative PNI might be an independent prognostic factor in BC patients after surgery, indicating that the evaluation of nutritional status and systemic immune should be integrated into the BC treatment. Further studies are required to explore the prognosis value of PLR.

## Supplementary Information

**Additional file 1:** **Supplementary Table 1.** Subgroup analyses in relation to tertiles of PNI with the DFS. **Supplementary Table 2.** Subgroup analyses in relation to tertiles of SII with the DFS. **Supplementary Table 3.** Subgroup analyses in relation to tertiles of NLR with the DFS. **Supplementary Table 4.** Subgroup analyses in relation to tertiles of PLR with the DFS. **Supplementary Figure 1.** The Kaplan-Meier disease-free survival curves of all patients in the cohort according to tertiles of SII (log-rank analysis *P *= 0.817). **Supplementary Figure 2.** The Kaplan-Meier disease-free survival curves of all patients in the cohort according to tertiles of NLR (log-rank analysis *P *= 0.600). **Supplementary Figure 3.** The Kaplan-Meier disease-free survival curves of all patients in the cohort according to tertiles of PLR (log-rank analysis *P *= 0.090).

## Data Availability

The datasets generated and analyzed during the current study are not publicly available due to its inclusion of health information, but are available from the corresponding author on reasonable request.

## Abbreviations

BC: Breast cancer
BMI: Body mass index
PNI: Prognostic nutritional index
SII: Systemic immune-inflammation index
NLR: Neutrophil–lymphocyte ratio
PLR: Platelet–lymphocyte ratio
DFS: Disease-free survival
OS: Overall survival
HR: Hazard ratio
DSS: Disease-specific survival
ER: Estrogen receptor
PR: Progesterone receptor
HER-2: Human epidermal growth factor receptor type 2
Ki67 index: Percentage of Ki67-positive cancer nuclei
LNM: Lymph node metastasis
IQR: Inter-quantile range
CA: Cochran-Armitage test
NK: Natural killer cells
