# Supplementary material for: Prognostic significance of prognostic nutritional index and systemic immune‐inflammation index in patients after curative breast cancer resection: a retrospective cohort study
Source: BMC Cancer. 2022 Nov 3;22:1128. doi: 10.1186/s12885-022-10218-x (PMC9632068; doi:10.1186/s12885-022-10218-x)
Supplement: Supplementary file 1 — Additional file 1: Supplementary Table 1. Subgroup analyses in relation to tertiles of PNI with the DFS. Supplementary Table 2. Subgroup analyses in relation to tertiles of SII with the DFS. Supplementary Table 3. Subgroup analyses in relation to tertiles of NLR with the DFS. Supplementary Table 4. Subgroup analyses in relation to tertiles of PLR with the DFS. Supplementary Figure 1. The Kaplan-Meier disease-free survival curves of all patients in the cohort according to tertiles of SII (log-rank analysis P = 0.817). Supplementary Figure 2. The Kaplan-Meier disease-free survival curves of all patients in the cohort according to tertiles of NLR (log-rank analysis P = 0.600). Supplementary Figure 3. The Kaplan-Meier disease-free survival curves of all patients in the cohort according to tertiles of PLR (log-rank analysis P = 0.090). [file 12885_2022_10218_MOESM1_ESM.docx]

**Prognostic significance of prognostic nutritional index and systemic immune‐inflammation index in patients after curative breast cancer resection: A retrospective cohort study**

**(Supplementary materials)**

**Table legends**

Supplementary Table 1. Subgroup analyses in relation to tertiles of PNI with the DFS.

Supplementary Table 2. Subgroup analyses in relation to tertiles of SII with the DFS.

Supplementary Table 3. Subgroup analyses in relation to tertiles of NLR with the DFS.

Supplementary Table 4. Subgroup analyses in relation to tertiles of PLR with the DFS.

**Figure legends**

Supplementary Figure.1 The Kaplan‐Meier disease-free survival curves of all patients in the cohort according to tertiles of SII (log-rank analysis *P* = 0.817).

Supplementary Figure.2 The Kaplan‐Meier disease-free survival curves of all patients in the cohort according to tertiles of NLR (log-rank analysis *P* = 0.600).

Supplementary Figure.3 The Kaplan‐Meier disease-free survival curves of all patients in the cohort according to tertiles of PLR (log-rank analysis *P* = 0.090).

**Supplementary Table 1.** Subgroup analyses in relation to tertiles of PNI with the DFS.

|  | **No.** | **T1** |  | **T2** | | | |  | **T3** | | | | ***P* interaction** |
| --- | --- | --- | --- | --- | --- | --- | --- | --- | --- | --- | --- | --- | --- |
|  |  | **HR** |  | **HR** | **95% CI** | | ***P* value** |  | **HR** | **95% CI** | | ***P* value** |  |
| **Family history of BC** |  |  |  |  |  |  |  |  |  |  |  |  | **0.004** |
| No | 367 | 1.00 (Ref) |  | 0.48 | 0.25 | 0.93 | 0.030 |  | 0.22 | 0.10 | 0.49 | <0.001 |  |
| Yes | 141 | 1.00 (Ref) |  | 0.98 | 0.24 | 4.07 | 0.979 |  | 1.65 | 0.51 | 5.32 | 0.399 |  |
| Body mass index (kg/m2) |  |  |  |  |  |  |  |  |  |  |  |  | 0.677 |
| 18.5-22.9 | 168 | 1.00 (Ref) |  | 0.68 | 0.21 | 2.24 | 0.527 |  | 0.11 | 0.02 | 0.58 | 0.009 |  |
| 23-27.4 | 236 | 1.00 (Ref) |  | 0.46 | 0.17 | 1.26 | 0.132 |  | 0.49 | 0.20 | 1.24 | 0.134 |  |
| ≥27.5 | 75 | 1.00 (Ref) |  | 0.21 | 0.03 | 1.50 | 0.120 |  | 0.31 | 0.06 | 1.69 | 0.175 |  |
| **Histological diagnosis** |  |  |  |  |  |  |  |  |  |  |  |  | 0.220 |
| Invasive carcinoma | 452 | 1.00 (Ref) |  | 0.52 | 0.27 | 0.99 | 0.047 |  | 0.44 | 0.23 | 0.83 | 0.011 |  |
| Carcinoma in situ | 56 | 1.00 (Ref) |  | 0.01 | 0.00 | 0.07 | <0.001 |  | 0.00 | 0.00 | Inf | 0.994 |  |
| **Clinical stage** |  |  |  |  |  |  |  |  |  |  |  |  | 0.347 |
| 0 | 53 | 1.00 (Ref) |  | Inf | 0 | Inf | 0.968 |  | Inf | 0 | Inf | 0.975 |  |
| I | 106 | 1.00 (Ref) |  | 0.12 | 0.01 | 1.33 | 0.085 |  | 0.26 | 0.06 | 1.24 | 0.091 |  |
| II | 243 | 1.00 (Ref) |  | 0.44 | 0.18 | 1.10 | 0.078 |  | 0.28 | 0.11 | 0.72 | 0.008 |  |
| III | 92 | 1.00 (Ref) |  | 0.71 | 0.22 | 2.34 | 0.575 |  | 0.37 | 0.10 | 1.34 | 0.129 |  |
| IV | 14 | 1.00 (Ref) |  | Inf | 0 | Inf | 1.000 |  | Inf | 0 | Inf | 1.000 |  |
| **Tumour size, cm** |  |  |  |  |  |  |  |  |  |  |  |  | 0.545 |
| ≥ 4 | 391 | 1.00 (Ref) |  | 0.45 | 0.22 | 0.92 | 0.028 |  | 0.37 | 0.19 | 0.73 | 0.004 |  |
| < 4 | 79 | 1.00 (Ref) |  | 0.59 | 0.10 | 3.55 | 0.564 |  | 0.09 | 0.01 | 1.27 | 0.075 |  |
| **Status of ER** |  |  |  |  |  |  |  |  |  |  |  |  | 0.890 |
| Negative | 149 | 1.00 (Ref) |  | 0.53 | 0.18 | 1.54 | 0.243 |  | 0.50 | 0.16 | 1.59 | 0.241 |  |
| Positive | 357 | 1.00 (Ref) |  | 0.56 | 0.28 | 1.10 | 0.091 |  | 0.41 | 0.20 | 0.84 | 0.015 |  |
| **Status of PR** |  |  |  |  |  |  |  |  |  |  |  |  | 0.625 |
| Negative | 203 | 1.00 (Ref) |  | 0.96 | 0.28 | 3.32 | 0.946 |  | 0.55 | 0.15 | 2.03 | 0.369 |  |
| Positive | 303 | 1.00 (Ref) |  | 0.54 | 0.26 | 1.09 | 0.083 |  | 0.42 | 0.20 | 0.88 | 0.021 |  |
| **Status of HER-2** |  |  |  |  |  |  |  |  |  |  |  |  | 0.984 |
| Negative | 114 | 1.00 (Ref) |  | 0.38 | 0.09 | 1.53 | 0.172 |  | 0.35 | 0.09 | 1.34 | 0.125 |  |
| Positive | 347 | 1.00 (Ref) |  |  |  |  |  |  |  |  |  |  | 0.529 |
| **Ki67-index** |  |  |  |  |  |  |  |  |  |  |  |  |  |
| ≥ 27.5% | 272 | 1.00 (Ref) |  | 0.70 | 0.29 | 1.69 | 0.424 |  | 0.42 | 0.17 | 1.05 | 0.063 |  |
| < 27.5% | 236 | 1.00 (Ref) |  | 0.41 | 0.16 | 1.10 | 0.078 |  | 0.31 | 0.11 | 0.83 | 0.020 |  |
| **Surgical forms** |  |  |  |  |  |  |  |  |  |  |  |  | 0.474 |
| Breast conserving surgery | 62 | 1.00 (Ref) |  | 0.10 | 0.02 | 0.53 | 0.007 |  | 0.04 | 0.00 | 0.32 | 0.003 |  |
| Modified radical mastectomy | 247 | 1.00 (Ref) |  | 0.70 | 0.31 | 1.57 | 0.385 |  | 0.48 | 0.20 | 1.15 | 0.100 |  |
| Total mastectomy | 188 | 1.00 (Ref) |  | 0.39 | 0.16 | 1.00 | 0.051 |  | 0.35 | 0.14 | 0.84 | 0.019 |  |
| Unclear | 11 | 1.00 (Ref) |  | NA | NA | NA | NA |  | NA | NA | NA | NA |  |
| **Chemotherapy** |  |  |  |  |  |  |  |  |  |  |  |  | 0.370 |
| No | 140 | 1.00 (Ref) |  | 0.20 | 0.05 | 0.79 | 0.021 |  | 0.11 | 0.02 | 0.60 | 0.011 |  |
| Yes | 368 | 1.00 (Ref) |  | 0.55 | 0.28 | 1.10 | 0.091 |  | 0.44 | 0.22 | 0.88 | 0.020 |  |
| **Radiotherapy** |  |  |  |  |  |  |  |  |  |  |  |  | **0.025** |
| No | 355 | 1.00 (Ref) |  | 0.55 | 0.26 | 1.15 | 0.113 |  | 0.63 | 0.31 | 1.27 | 0.197 |  |
| Yes | 368 | 1.00 (Ref) |  | 0.55 | 0.28 | 1.10 | 0.092 |  | 0.45 | 0.23 | 0.88 | 0.020 |  |
| **Endocrine therapy** |  |  |  |  |  |  |  |  |  |  |  |  | 0.742 |
| No | 160 | 1.00 (Ref) |  | 1.58 | 0.40 | 6.26 | 0.516 |  | 0.93 | 0.20 | 4.30 | 0.922 |  |
| Yes | 348 | 1.00 (Ref) |  | 0.48 | 0.24 | 0.95 | 0.035 |  | 0.38 | 0.19 | 0.77 | 0.007 |  |
| **Targeted therapy** |  |  |  |  |  |  |  |  |  |  |  |  | 0.996 |
| No | 462 | 1.00 (Ref) |  | 0.57 | 0.31 | 1.02 | 0.059 |  | 0.41 | 0.22 | 0.76 | 0.004 |  |
| Yes | 46 | 1.00 (Ref) |  | 0.00 | 0.00 | Inf | 1.000 |  | 0.00 | 0.00 | Inf | 1.000 |  |
| **LNM, clinical** |  |  |  |  |  |  |  |  |  |  |  |  | 0.613 |
| No | 283 | 1.00 (Ref) |  | 0.34 | 0.15 | 0.79 | 0.012 |  | 0.32 | 0.14 | 0.72 | 0.006 |  |
| Yes | 223 | 1.00 (Ref) |  | 0.82 | 0.35 | 1.90 | 0.638 |  | 0.37 | 0.14 | 0.99 | 0.048 |  |

Notes: All the data were adjusted for the covariates in multivariate model; *P* for trend, *P* value for trend across tertiles; *P* for interaction, *P* value for interaction across variable; T1-T3, PNI were divided into 3 quartiles: ≤ 53.0, 53.0~57.5, >57.5;

Abbreviations: BC, breast cancer; PNI, prognostic nutritional index; DFS, disease free-survival; ER, estrogen receptor; PR, progesterone receptor; HER-2, human epidermal growth factor receptor type 2; Ki67 index, percentage of Ki67-positive cancer nuclei; LNM, lymph node metastasis; Inf: infinite; NA: not available.

**Supplementary Table 2.** Subgroup analyses in relation to tertiles of SII with the DFS.

|  | **No.** | **T1** |  | **T2** | | | |  | **T3** | | | | ***P* interaction** |
| --- | --- | --- | --- | --- | --- | --- | --- | --- | --- | --- | --- | --- | --- |
|  |  | **HR** |  | **HR** | **95% CI** | | ***P* value** |  | **HR** | **95% CI** | | ***P* value** |  |
| **Family history of BC** |  |  |  |  |  |  |  |  |  |  |  |  | 0.178 |
| No | 367 | 1.00 (Ref) |  | 1.26 | 0.64 | 2.51 | 0.506 |  | 0.72 | 0.33 | 1.55 | 0.400 |  |
| Yes | 141 | 1.00 (Ref) |  | 7.15 | 1.40 | 36.52 | 0.018 |  | 2.12 | 0.33 | 13.40 | 0.426 |  |
| **Body mass index (kg/m2)** |  |  |  |  |  |  |  |  |  |  |  |  | 0.480 |
| 18.5-22.9 | 168 | 1.00 (Ref) |  | 3.93 | 1.15 | 13.43 | 0.029 |  | 0.83 | 0.17 | 3.94 | 0.810 |  |
| 23-27.4 | 236 | 1.00 (Ref) |  | 1.09 | 0.45 | 2.67 | 0.846 |  | 0.62 | 0.22 | 1.75 | 0.364 |  |
| ≥27.5 | 75 | 1.00 (Ref) |  | 2.30 | 0.36 | 14.76 | 0.379 |  | 1.47 | 0.16 | 13.19 | 0.729 |  |
| **Histological diagnosis** |  |  |  |  |  |  |  |  |  |  |  |  | 0.849 |
| Invasive carcinoma | 452 | 1.00 (Ref) |  | 1.75 | 0.92 | 3.33 | 0.087 |  | 0.93 | 0.45 | 1.96 | 0.858 |  |
| Carcinoma in situ | 56 | 1.00 (Ref) |  | 0.77 | 0.14 | 4.37 | 0.771 |  | 0.23 | 0.04 | 1.40 | 0.111 |  |
| **Clinical stage** |  |  |  |  |  |  |  |  |  |  |  |  | 0.401 |
| 0 | 53 | 1.00 (Ref) |  | Inf | Inf | Inf | <0.001 |  | 0.27 | 0.02 | 3.15 | 0.295 |  |
| I | 106 | 1.00 (Ref) |  | 2.63 | 0.55 | 12.57 | 0.226 |  | 0.83 | 0.14 | 4.90 | 0.841 |  |
| II | 243 | 1.00 (Ref) |  | 2.47 | 0.95 | 6.46 | 0.065 |  | 1.52 | 0.52 | 4.38 | 0.442 |  |
| III | 92 | 1.00 (Ref) |  | 0.96 | 0.29 | 3.19 | 0.950 |  | 0.56 | 0.10 | 3.12 | 0.505 |  |
| IV | 14 | 1.00 (Ref) |  | Inf | 0 | Inf | 1.000 |  | Inf | 0 | Inf | 1.000 |  |
| **Tumour size, cm** |  |  |  |  |  |  |  |  |  |  |  |  | 0.241 |
| ≥ 4 | 391 | 1.00 (Ref) |  | 1.80 | 0.90 | 3.57 | 0.094 |  | 0.79 | 0.35 | 1.74 | 0.554 |  |
| < 4 | 79 | 1.00 (Ref) |  | 0.09 | 0.01 | 1.15 | 0.064 |  | 1.45 | 0.15 | 14.59 | 0.750 |  |
| **Status of ER** |  |  |  |  |  |  |  |  |  |  |  |  | 0.579 |
| Negative | 149 | 1.00 (Ref) |  | 3.47 | 1.26 | 9.56 | 0.016 |  | 2.55 | 0.81 | 8.03 | 0.111 |  |
| Positive | 357 | 1.00 (Ref) |  | 1.45 | 0.73 | 2.87 | 0.292 |  | 0.81 | 0.37 | 1.77 | 0.590 |  |
| **Status of PR** |  |  |  |  |  |  |  |  |  |  |  |  | 0.917 |
| Negative | 203 | 1.00 (Ref) |  | 2.07 | 0.64 | 6.76 | 0.227 |  | 1.04 | 0.22 | 5.00 | 0.960 |  |
| Positive | 303 | 1.00 (Ref) |  | 1.57 | 0.75 | 3.28 | 0.226 |  | 0.93 | 0.42 | 2.08 | 0.868 |  |
| **Status of HER-2** |  |  |  |  |  |  |  |  |  |  |  |  | 0.396 |
| Negative | 114 | 1.00 (Ref) |  | 0.64 | 0.20 | 2.03 | 0.451 |  | 0.30 | 0.07 | 1.36 | 0.118 |  |
| Positive | 347 | 1.00 (Ref) |  | 2.38 | 1.05 | ,5.38 | 0.038 |  | 0.92 | 0.35 | 2.40 | 0.861 |  |
| **Ki67-index** |  |  |  |  |  |  |  |  |  |  |  |  | 0.391 |
| ≥ 27.5% | 272 | 1.00 (Ref) |  | 1.31 | 0.58 | 2.92 | 0.516 |  | 0.71 | 0.25 | 1.97 | 0.508 |  |
| < 27.5% | 236 | 1.00 (Ref) |  | 3.29 | 1.05 | 10.31 | 0.041 |  | 1.48 | 0.41 | 5.34 | 0.545 |  |
| **Surgical forms** |  |  |  |  |  |  |  |  |  |  |  |  | 0.560 |
| Breast conserving surgery | 62 | 1.00 (Ref) |  | 2.67 | 0.50 | 14.15 | 0.248 |  | 1.18 | 0.22 | 6.33 | 0.846 |  |
| Modified radical mastectomy | 247 | 1.00 (Ref) |  | 1.80 | 0.75 | 4.30 | 0.185 |  | 1.54 | 0.57 | 4.19 | 0.185 |  |
| Total mastectomy | 188 | 1.00 (Ref) |  | 1.67 | 0.75 | 3.73 | 0.213 |  | 0.82 | 0.33 | 2.08 | 0.683 |  |
| Unclear | 11 | 1.00 (Ref) |  | NA | NA | NA | NA |  | NA | NA | NA | NA |  |
| **Chemotherapy** |  |  |  |  |  |  |  |  |  |  |  |  | 0.453 |
| No | 140 | 1.00 (Ref) |  | 3.57 | 0.81 | 15.62 | 0.091 |  | 1.02 | 0.19 | 5.48 | 0.986 |  |
| Yes | 368 | 1.00 (Ref) |  | 1.50 | 0.77 | 2.93 | 0.238 |  | 0.83 | 0.38 | 1.81 | 0.634 |  |
| **Radiotherapy** |  |  |  |  |  |  |  |  |  |  |  |  | 0.411 |
| No | 355 | 1.00 (Ref) |  | 1.57 | 0.76 | 3.25 | 0.224 |  | 1.09 | 0.49 | 2.41 | 0.836 |  |
| Yes | 368 | 1.00 (Ref) |  | 1.49 | 0.76 | 2.92 | 0.241 |  | 0.83 | 0.38 | 1.80 | 0.632 |  |
| **Endocrine therapy** |  |  |  |  |  |  |  |  |  |  |  |  | 0.333 |
| No | 160 | 1.00 (Ref) |  | 3.84 | 0.73 | 20.18 | 0.112 |  | 2.45 | 0.42 | 14.42 | 0.321 |  |
| Yes | 348 | 1.00 (Ref) |  | 1.53 | 0.77 | 3.03 | 0.227 |  | 0.80 | 0.36 | 1.80 | 0.591 |  |
| **Targeted therapy** |  |  |  |  |  |  |  |  |  |  |  |  | 1.000 |
| No | 462 | 1.00 (Ref) |  | 1.59 | 0.88 | 2.88 | 0.128 |  | 0.88 | 0.44 | 1.74 | 0.711 |  |
| Yes | 46 | 1.00 (Ref) |  | Inf | 0.00 | Inf | 1.000 |  | Inf | 0.00 | Inf | 1.000 |  |
| **LNM, clinical** |  |  |  |  |  |  |  |  |  |  |  |  | 0.542 |
| No | 283 | 1.00 (Ref) |  | 1.60 | 0.72 | 3.57 | 0.250 |  | 0.72 | 0.29 | 1.77 | 0.473 |  |
| Yes | 223 | 1.00 (Ref) |  | 1.76 | 0.68 | 4.59 | 0.245 |  | 1.30 | 0.44 | 3.83 | 0.635 |  |

Notes: All the data were adjusted for the covariates in multivariate model; *P* for trend, *P* value for trend across tertiles; *P* for interaction, *P* value for interaction across variable; T1-T3, SII were divided into 3 tertiles: ≤ 429.43, 429.43~665.24, >665.24;

Abbreviations: BC, breast cancer; SII, systemic immune‐inflammation index; DFS, disease free-survival; ER, estrogen receptor; PR, progesterone receptor; HER-2, human epidermal growth factor receptor type 2; Ki67 index, percentage of Ki67-positive cancer nuclei; LNM, lymph node metastasis; Inf: infinite; NA: not available.

**Supplementary Table 3.** Subgroup analyses in relation to tertiles of NLR with the DFS.

|  | **No.** | **T1** |  | **T2** | | | |  | **T3** | | | | ***P* interaction** |
| --- | --- | --- | --- | --- | --- | --- | --- | --- | --- | --- | --- | --- | --- |
|  |  | **HR** |  | **HR** | **95% CI** | | ***P* value** |  | **HR** | **95% CI** | | ***P* value** |  |
| **Family history of BC** |  |  |  |  |  |  |  |  |  |  |  |  | 0.205 |
| No | 367 | 1.00 (Ref) |  | 1.16 | 0.57 | 2.39 | 0.681 |  | 1.05 | 0.50 | 2.20 | 0.897 |  |
| Yes | 141 | 1.00 (Ref) |  | 1.21 | 0.26 | 5.60 | 0.812 |  | 2.63 | 0.71 | 9.69 | 0.147 |  |
| **Body mass index (kg/m2)** |  |  |  |  |  |  |  |  |  |  |  |  | 0.093 |
| 18.5-22.9 | 168 | 1.00 (Ref) |  | 1.91 | 0.55 | 6.58 | 0.308 |  | 1.24 | 0.35 | 4.44 | 0.737 |  |
| 23-27.4 | 236 | 1.00 (Ref) |  | 1.12 | 0.43 | 2.96 | 0.813 |  | 1.05 | 0.40 | 2.74 | 0.927 |  |
| ≥27.5 | 75 | 1.00 (Ref) |  | 5.35 | 0.67 | 42.92 | 0.114 |  | 6.99 | 0.91 | 53.66 | 0.062 |  |
| **Histological diagnosis** |  |  |  |  |  |  |  |  |  |  |  |  | 0.483 |
| Invasive carcinoma | 452 | 1.00 (Ref) |  | 1.21 | 0.62 | 2.34 | 0.578 |  | 1.33 | 0.69 | 2.58 | 0.395 |  |
| Carcinoma in situ | 56 | 1.00 (Ref) |  | 0.76 | 0.09 | 6.64 | 0.803 |  | 0.85 | 0.15 | 4.70 | 0.849 |  |
| **Clinical stage** |  |  |  |  |  |  |  |  |  |  |  |  | 0.196 |
| 0 | 53 | 1.00 (Ref) |  | Inf | 0 | Inf | 0.987 |  | 0.58 | 0.04 | 9.39 | 0.070 |  |
| I | 106 | 1.00 (Ref) |  | 1.01 | 0.19 | 5.40 | 0.988 |  | 1.43 | 0.29 | 6.97 | 0.658 |  |
| II | 243 | 1.00 (Ref) |  | 3.01 | 0.98 | 9.28 | 0.054 |  | 3.06 | 1.04 | 9.02 | 0.042 |  |
| III | 92 | 1.00 (Ref) |  | 0.52 | 0.16 | 1.66 | 0.267 |  | 0.71 | 0.19 | 2.63 | 0.612 |  |
| IV | 14 | 1.00 (Ref) |  | 0.96 | 0 | Inf | 1.000 |  | 0.96 | 0 | Inf | 1.000 |  |
| **Tumour size, cm** |  |  |  |  |  |  |  |  |  |  |  |  | 0.821 |
| ≥ 27.5% | 391 | 1.00 (Ref) |  | 1.29 | 0.63 | 2.65 | 0.480 |  | 1.21 | 0.59 | 2.47 | 0.597 |  |
| < 27.5% | 79 | 1.00 (Ref) |  | 3.49 | 0.24 | 51.53 | 0.364 |  | 4.29 | 0.29 | 63.85 | 0.290 |  |
| **Status of ER** |  |  |  |  |  |  |  |  |  |  |  |  | 0.568 |
| Negative | 149 | 1.00 (Ref) |  | 1.67 | 0.53 | 5.25 | 0.383 |  | 2.78 | 1.03 | 7.55 | 0.044 |  |
| Positive | 357 | 1.00 (Ref) |  | 1.20 | 0.58 | 2.46 | 0.628 |  | 1.22 | 0.58 | 2.56 | 0.602 |  |
| **Status of PR** |  |  |  |  |  |  |  |  |  |  |  |  | 0.892 |
| Negative | 203 | 1.00 (Ref) |  | 1.36 | 0.37 | 5.08 | 0.644 |  | 1.44 | 0.38 | 5.45 | 0.590 |  |
| Positive | 303 | 1.00 (Ref) |  | 1.26 | 0.58 | 2.74 | 0.564 |  | 1.41 | 0.66 | 3.01 | 0.374 |  |
| **Status of HER-2** |  |  |  |  |  |  |  |  |  |  |  |  | 0.693 |
| Negative | 114 | 1.00 (Ref) |  | 0.72 | 0.18 | 2.83 | 0.639 |  | 0.41 | 0.10 | 1.69 | 0.217 |  |
| Positive | 347 | 1.00 (Ref) |  | 1.82 | 0.74 | 4.47 | 0.195 |  | 2.11 | 0.88 | 5.0 | 0.095 |  |
| **Ki67-index** |  |  |  |  |  |  |  |  |  |  |  |  | 0.113 |
| ≥ 4 | 272 | 1.00 (Ref) |  | 1.08 | 0.46 | 2.56 | 0.859 |  | 1.01 | 0.39 | 2.58 | 0.991 |  |
| < 4 | 236 | 1.00 (Ref) |  | 2.81 | 0.81 | 9.80 | 0.105 |  | 3.31 | 1.02 | 10.74 | 0.046 |  |
| **Surgical forms** |  |  |  |  |  |  |  |  |  |  |  |  | 0.810 |
| Breast conserving surgery | 62 | 1.00 (Ref) |  | 1.68 | 0.33 | 9.85 | 0.845 |  | 2.11 | 0.47 | 9.53 | 0.333 |  |
| Modified radical mastectomy | 247 | 1.00 (Ref) |  | 0.95 | 0.38 | 2.35 | 0.904 |  | 1.95 | 0.86 | 4.45 | 0.112 |  |
| Total mastectomy | 188 | 1.00 (Ref) |  | 2.89 | 1.28 | 6.49 | 0.010 |  | 1.42 | 0.58 | 3.46 | 0.440 |  |
| Unclear | 11 | 1.00 (Ref) |  | NA | NA | NA | NA |  | NA | NA | NA | NA |  |
| **Chemotherapy** |  |  |  |  |  |  |  |  |  |  |  |  | 0.809 |
| No | 140 | 1.00 (Ref) |  | 2.68 | 0.59 | 12.10 | 0.199 |  | 1.28 | 0.25 | 6.58 | 0.766 |  |
| Yes | 368 | 1.00 (Ref) |  | 1.06 | 0.51 | 2.23 | 0.872 |  | 1.41 | 0.70 | 2.84 | 0.334 |  |
| **Radiotherapy** |  |  |  |  |  |  |  |  |  |  |  |  | 0.678 |
| No | 355 | 1.00 (Ref) |  | 1.74 | 0.83 | 3.67 | 0.145 |  | 1.19 | 0.55 | 2.60 | 0.660 |  |
| Yes | 368 | 1.00 (Ref) |  | 1.06 | 0.51 | 2.22 | 0.873 |  | 1.41 | 0.70 | 2.83 | 0.333 |  |
| **Endocrine therapy** |  |  |  |  |  |  |  |  |  |  |  |  | 0.754 |
| No | 160 | 1.00 (Ref) |  | 1.39 | 0.27 | 7.16 | 0.693 |  | 1.61 | 0.39 | 6.58 | 0.509 |  |
| Yes | 348 | 1.00 (Ref) |  | 1.32 | 0.63 | 2.77 | 0.462 |  | 1.41 | 0.67 | 2.97 | 0.365 |  |
| **Targeted therapy** |  |  |  |  |  |  |  |  |  |  |  |  | 1.000 |
| No | 462 | 1.00 (Ref) |  | 1.26 | 0.66 | 2.38 | 0.484 |  | 1.34 | 0.72 | 2.50 | 0.354 |  |
| Yes | 46 | 1.00 (Ref) |  | Inf | 0.00 | Inf | 1.000 |  | Inf | 0.00 | Inf | 1.000 |  |
| **LNM, clinical** |  |  |  |  |  |  |  |  |  |  |  |  | 0.932 |
| No | 283 | 1.00 (Ref) |  | 2.07 | 0.83 | 5.15 | 0.116 |  | 1.42 | 0.57 | 3.55 | 0.447 |  |
| Yes | 223 | 1.00 (Ref) |  | 0.70 | 0.26 | 1.88 | 0.484 |  | 1.49 | 0.61 | 3.63 | 0.384 |  |

Notes: All the data were adjusted for the covariates in multivariate model; *P* for trend, *P* value for trend across tertiles; *P* for interaction, *P* value for interaction across variable; T1-T3, NLR were divided into 3 tertiles: ≤ 1.71, 1.71~2.43, >2.43;

Abbreviations: BC, breast cancer; NLR, neutrophil-lymphocyte ratio; DFS, disease free-survival; ER, estrogen receptor; PR, progesterone receptor; HER-2, human epidermal growth factor receptor type 2; Ki67 index, percentage of Ki67-positive cancer nuclei; LNM, lymph node metastasis; Inf: infinite; NA: not available.

**Supplementary Table 4.** Subgroup analyses in relation to tertiles of PLR with the DFS.

|  | **No.** | **T1** |  | **T2** | | | |  | **T3** | | | | ***P* interaction** |
| --- | --- | --- | --- | --- | --- | --- | --- | --- | --- | --- | --- | --- | --- |
|  |  | **HR** |  | **HR** | **95% CI** | | ***P* value** |  | **HR** | **95% CI** | | ***P* value** |  |
| **Family history of BC** |  |  |  |  |  |  |  |  |  |  |  |  | 0.713 |
| No | 367 | 1.00 (Ref) |  | 1.61 | 0.75 | 3.45 | 0.218 |  | 1.63 | 0.75 | 3.55 | 0.220 |  |
| Yes | 141 | 1.00 (Ref) |  | 3.94 | 0.95 | 16.33 | 0.059 |  | 1.58 | 0.33 | 7.64 | 0.567 |  |
| **Body mass index (kg/m2)** |  |  |  |  |  |  |  |  |  |  |  |  | 0.126 |
| 18.5-22.9 | 168 | 1.00 (Ref) |  | 11.08 | 1.84 | 63.31 | 0.008 |  | 4.71 | 0.73 | 30.53 | 0.104 |  |
| 23-27.4 | 236 | 1.00 (Ref) |  | 1.79 | 0.71 | 4.50 | 0.219 |  | 1.18 | 0.44 | 3.16 | 0.736 |  |
| ≥27.5 | 75 | 1.00 (Ref) |  | 0.94 | 0.07 | 13.0 | 0.963 |  | 9.36 | 1.45 | 60.16 | 0.019 |  |
| **Surgical forms** |  |  |  |  |  |  |  |  |  |  |  |  | 0.369 |
| Breast conserving surgery | 62 | 1.00 (Ref) |  | 10.18 | 2.01 | 51.55 | 0.005 |  | 1.79 | 0.21 | 15.62 | 0.598 |  |
| Modified radical mastectomy | 247 | 1.00 (Ref) |  | 1.78 | 0.67 | 4.75 | 0.247 |  | 2.91 | 1.14 | 7.42 | 0.025 |  |
| Total mastectomy | 188 | 1.00 (Ref) |  | 1.85 | 0.83 | 4.12 | 0.135 |  | 1.81 | 0.75 | 4.40 | 0.190 |  |
| Unclear | 11 | 1.00 (Ref) |  | NA | NA | NA | NA |  | NA | NA | NA | NA |  |
| **Histological diagnosis** |  |  |  |  |  |  |  |  |  |  |  |  | 0.165 |
| Invasive carcinoma | 452 | 1.00 (Ref) |  | 2.56 | 1.25 | 5.28 | 0.011 |  | 2.13 | 1.01 | 4.49 | 0.048 |  |
| Carcinoma in situ | 56 | 1.00 (Ref) |  | 6.93 | 1.33 | 35.94 | 0.021 |  | 4.96 | 0.55 | 45.06 | 0.155 |  |
| **Clinical stage** |  |  |  |  |  |  |  |  |  |  |  |  | 0.555 |
| 0 | 53 | 1.00 (Ref) |  | Inf | 0 | Inf | 0.911 |  | Inf | 0 | Inf | 0.973 |  |
| I | 106 | 1.00 (Ref) |  | 2.06 | 0.38 | 11.08 | 0.401 |  | 1.20 | 0.25 | 5.77 | 0.824 |  |
| II | 243 | 1.00 (Ref) |  | 4.61 | 1.49 | 14.26 | 0.008 |  | 3.82 | 1.13 | 12.83 | 0.030 |  |
| III | 92 | 1.00 (Ref) |  | 0.97 | 0.26 | 3.57 | 0.963 |  | 1.80 | 0.49 | 6.66 | 0.376 |  |
| IV | 14 | 1.00 (Ref) |  | NA | NA | NA | NA |  | NA | NA | NA | NA |  |
| **Tumour size, cm** |  |  |  |  |  |  |  |  |  |  |  |  | 0.445 |
| ≥ 4 | 391 | 1.00 (Ref) |  | 3.05 | 1.34 | 6.95 | 0.008 |  | 2.42 | 1.03 | 5.70 | 0.043 |  |
| < 4 | 79 | 1.00 (Ref) |  | 0.37 | 0.04 | 3.67 | 0.395 |  | 1.20 | 0.20 | 7.25 | 0.840 |  |
| **Status of ER** |  |  |  |  |  |  |  |  |  |  |  |  | 0.861 |
| Negative | 149 | 1.00 (Ref) |  | 1.42 | 0.53 | 3.85 | 0.487 |  | 1.60 | 0.55 | 4.64 | 0.390 |  |
| Positive | 357 | 1.00 (Ref) |  | 2.12 | 0.98 | 4.62 | 0.057 |  | 1.85 | 0.83 | 4.14 | 0.134 |  |
| **Status of PR** |  |  |  |  |  |  |  |  |  |  |  |  | 0.579 |
| Negative | 203 | 1.00 (Ref) |  | 2.21 | 0.65 | 7.51 | 0.206 |  | 1.64 | 0.37 | 7.25 | 0.516 |  |
| Positive | 303 | 1.00 (Ref) |  | 1.95 | 0.87 | 4.36 | 0.104 |  | 1.96 | 0.87 | 4.43 | 0.107 |  |
| **Chemotherapy** |  |  |  |  |  |  |  |  |  |  |  |  | 0.937 |
| No | 140 | 1.00 (Ref) |  | 0.91 | 0.18 | 4.61 | 0.913 |  | 1.73 | 0.45 | 6.58 | 0.422 |  |
| Yes | 368 | 1.00 (Ref) |  | 3.16 | 1.45 | 6.90 | 0.004 |  | 2.00 | 0.87 | 4.59 | 0.103 |  |
| **Radiotherapy** |  |  |  |  |  |  |  |  |  |  |  |  | 0.501 |
| No | 355 | 1.00 (Ref) |  | 1.76 | 0.80 | 3.90 | 0.162 |  | 1.91 | 0.86 | 4.26 | 0.114 |  |
| Yes | 368 | 1.00 (Ref) |  | 3.16 | 1.45 | 6.89 | 0.004 |  | 1.99 | 0.87 | 4.58 | 0.104 |  |
| **Status of HER-2** |  |  |  |  |  |  |  |  |  |  |  |  | 0.377 |
| Negative | 114 | 1.00 (Ref) |  | 2.35 | 0.63 | 8.74 | 0.201 |  | 1.84 | 0.43 | 7.88 | 0.411 |  |
| Positive | 347 | 1.00 (Ref) |  | 2.26 | 0.94 | 5.46 | 0.070 |  | 1.77 | 0.70 | 4.45 | 0.227 |  |
| **Ki67-index** |  |  |  |  |  |  |  |  |  |  |  |  | 0.737 |
| ≥ 27.5% | 272 | 1.00 (Ref) |  | 3.00 | 1.18 | 7.65 | 0.021 |  | 1.92 | 0.72 | 5.11 | 0.194 |  |
| < 27.5% | 236 | 1.00 (Ref) |  | 2.33 | 0.75 | 7.23 | 0.145 |  | 2.64 | 0.82 | 8.52 | 0.104 |  |
| **Endocrine therapy** |  |  |  |  |  |  |  |  |  |  |  |  | 0.965 |
| No | 160 | 1.00 (Ref) |  | 1.88 | 0.42 | 8.39 | 0.410 |  | 2.00 | 0.46 | 8.79 | 0.357 |  |
| Yes | 348 | 1.00 (Ref) |  | 2.25 | 1.04 | 4.86 | 0.039 |  | 1.85 | 0.82 | 4.16 | 0.137 |  |
| **Targeted therapy** |  |  |  |  |  |  |  |  |  |  |  |  | 0.911 |
| No | 462 | 1.00 (Ref) |  | 1.95 | 1.01 | 3.77 | 0.047 |  | 1.72 | 0.87 | 3.42 | 0.121 |  |
| Yes | 46 | 1.00 (Ref) |  | Inf | 0.00 | Inf | 1.000 |  | Inf | 0.00 | Inf | 1.000 |  |
| **LNM, clinical** |  |  |  |  |  |  |  |  |  |  |  |  | 0.355 |
| No | 283 | 1.00 (Ref) |  | 1.82 | 0.78 | 4.25 | 0.163 |  | 1.19 | 0.47 | 3.02 | 0.718 |  |
| Yes | 223 | 1.00 (Ref) |  | 2.49 | 0.81 | 7.43 | 0.101 |  | 3.32 | 1.11 | 9.97 | 0.032 |  |

Notes: All the data were adjusted for the covariates in multivariate model; *P* for trend, *P* value for trend across tertiles; *P* for interaction, *P* value for interaction across variable; T1-T3, PLR were divided into 3 tertiles: ≤ 115.66, 115.66~150.65, >150.65;

Abbreviations: BC, breast cancer; PLR, platelet-lymphocyte ratio; DFS, disease free-survival; ER, estrogen receptor; PR, progesterone receptor; HER-2, human epidermal growth factor receptor type 2; Ki67 index, percentage of Ki67-positive cancer nuclei; LNM, lymph node metastasis; Inf: infinite; NA: not available.


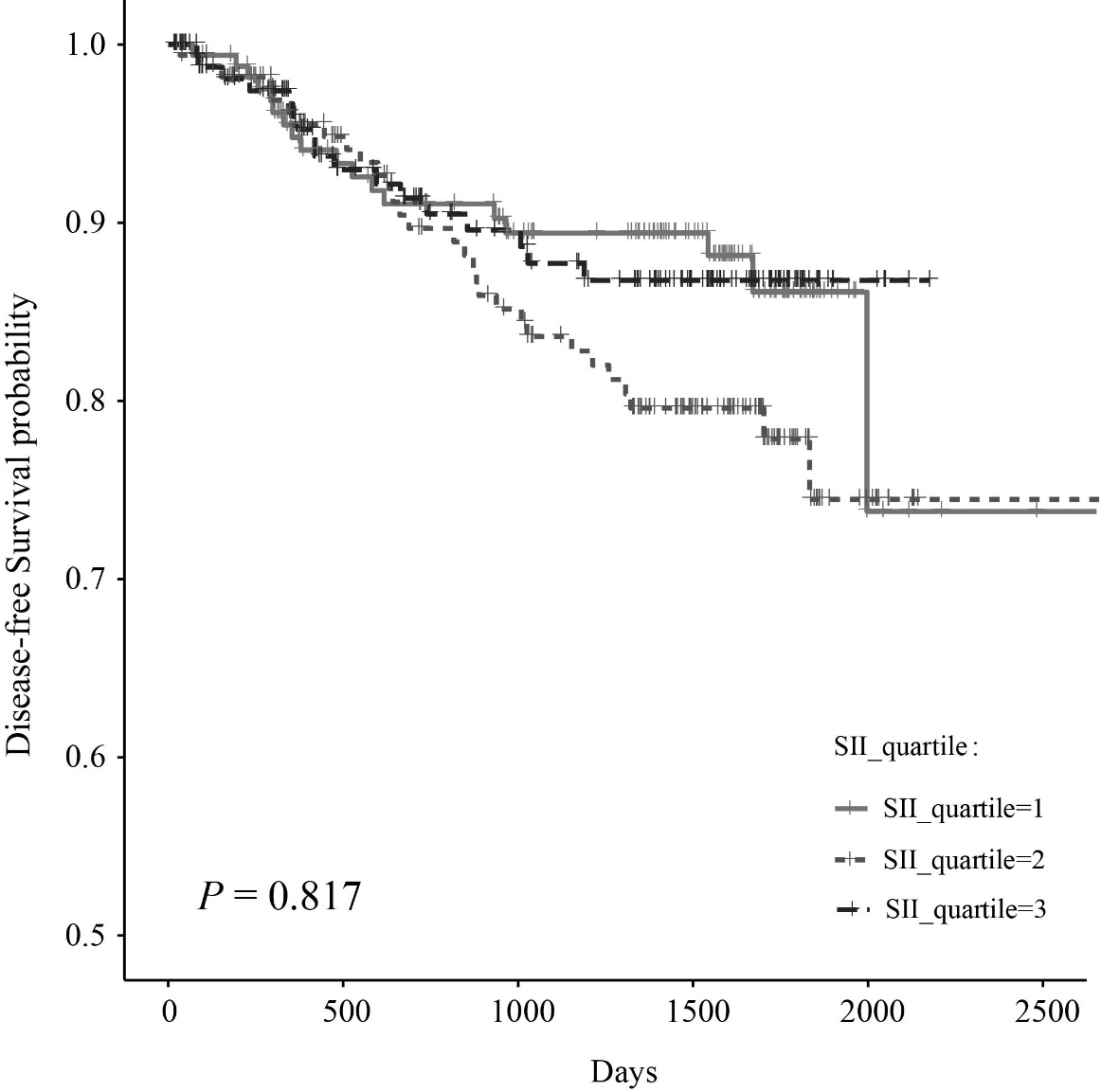


**Supplementary Figure.1** Kaplan‐Meier disease-free survival curves of all patients in the cohort according to tertiles of SII (log-rank analysis *P* = 0.817).


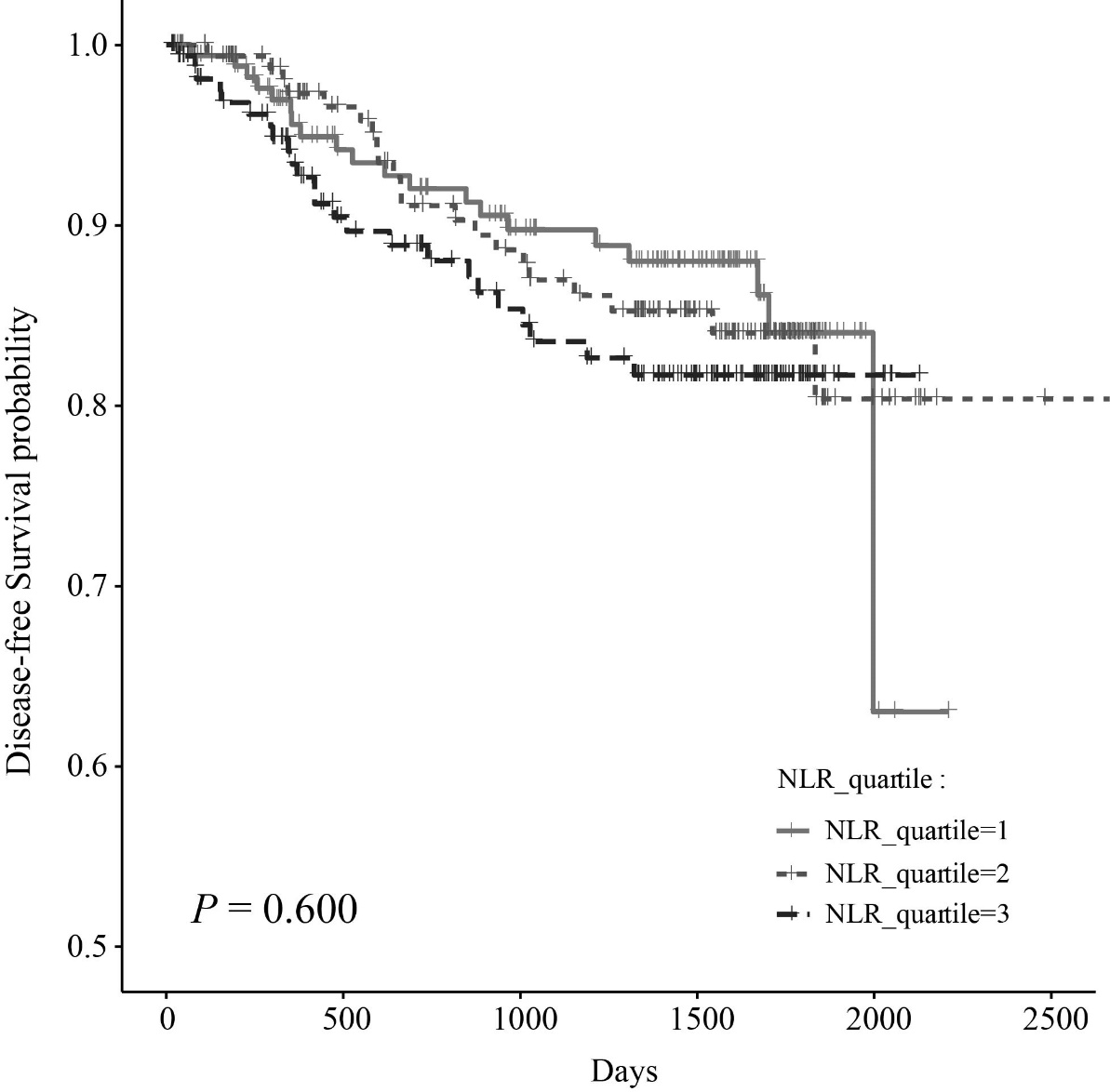


**Supplementary Figure.2** Kaplan‐Meier disease-free survival curves of all patients in the cohort according to tertiles of NLR (log-rank analysis *P* = 0.600).


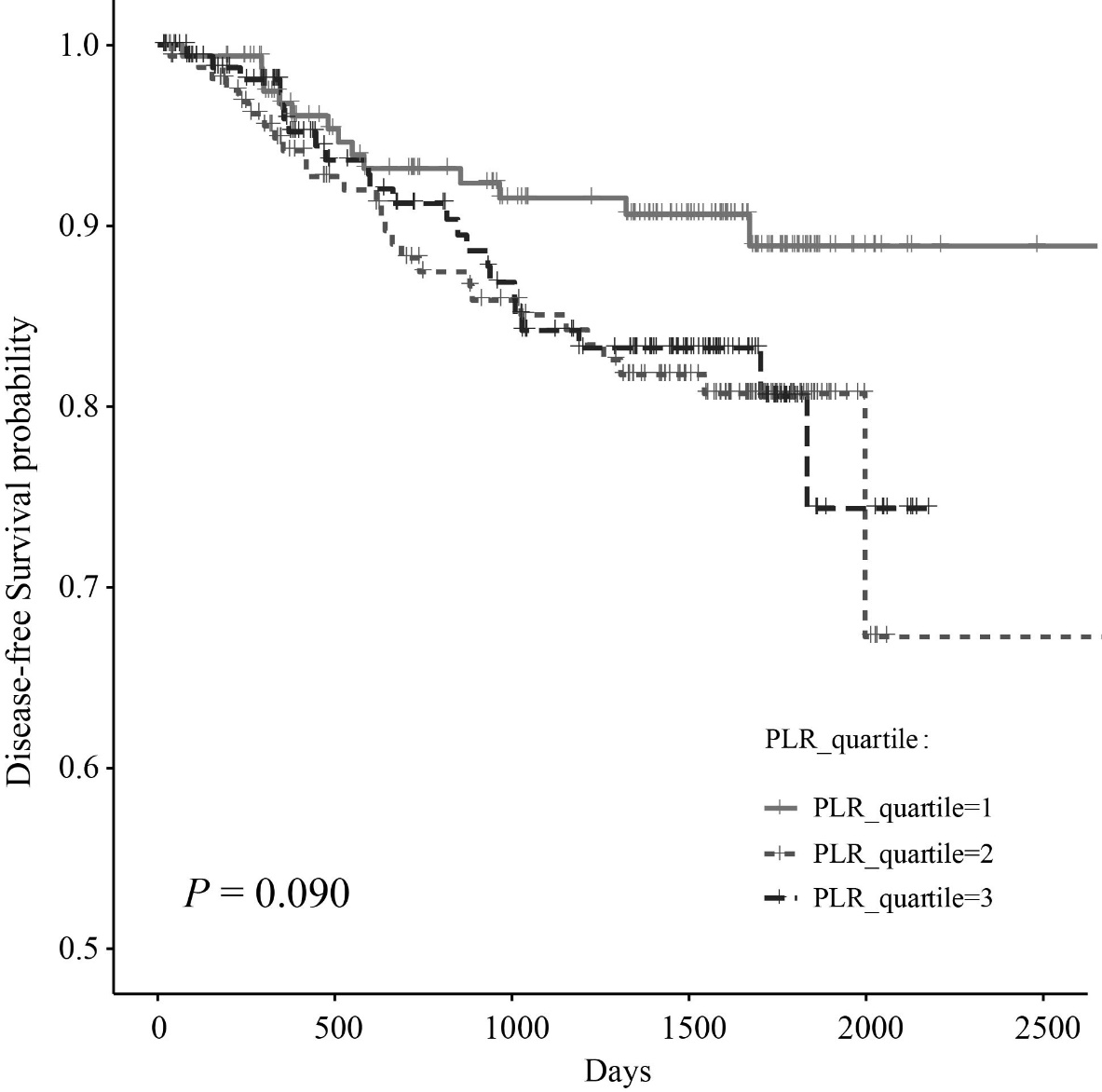


**Supplementary Figure.3** Kaplan‐Meier disease-free survival curves of all patients in the cohort according to tertiles of PLR (log-rank analysis *P* = 0.090).
